# Machine learning-assisted identification of predictive biomarkers for latent liability to schizotypy and bipolarity

**DOI:** 10.1192/j.eurpsy.2026.11838

**Published:** 2026-07-31

**Authors:** S. Finlay, G. Voss, O. Adegboye, D. Rudd, B. McDermott, D. Kata, I. Földesi, M. R. Azghadi, Z. Sarnyai, I. Szendi

**Affiliations:** 1 Australian Institute of Tropical Health and Medicine; 2 College of Medicine and Dentistry; 3 Margaret Roderick Centre for Mental Health Research; 4 College of Science and Engineering, James Cook University, Townsville; 5 Menzies School of Health Research, Charles Darwin University, Casuarina; 6 Child and Adolescent Mental Health Service; 7 Tasmanian Centre for Mental Health Service Innovation, Hobart, Australia; 8 Institute of Laboratory Medicine, University of Szeged, Szeged; 9 Psychiatry, Kiskunhalas Semmelweis University Teaching Hospital, Kiskunhalas; 10 Institute of Psychology; 11 Centre of Excellence for Interdisciplinary Research, Development and Innovation, University of Szeged, Szeged, Hungary

## Abstract

**Introduction:**

Psychotic disorders often emerge from subclinical traits linked to schizotypy or bipolarity, and the crucial selective or indicated prevention could be implemented based on the identification of these.

**Objectives:**

This study identifies low-risk individuals in a non-clinical population using psychometric measures, and our goal was to explore allostatic load (AL) as a marker of latent vulnerability.

**Methods:**

Three groups were created based on psychometric measures, which identified 30 individuals with positive schizotypy traits (PSF), 25 with cyclothymic bipolarity traits (CF), and 30 healthy controls (HCs). Allostatic load was calculated using 21 biomarkers, and Machine learning (ML) models identified key predictive features and reduced-feature models validated robustness.

**Results:**

Significant differences were observed between the three groups in the oxidative system (*p* = 0.014). SHAP analysis revealed creatinine, diastolic blood pressure, uric acid, and low-density lipids as key predictors for the CF group and heart rate, TSH, uric acid, and glucose for the PSF group. Machine learning achieved 76% accuracy using all 21 biomarkers and maintained the performance using a reduced 10-biomarker model. The AL was significantly (*p* = 0.010 and 0.005) lower in the CF individuals than HCs using 10 and 6 biomarkers, respectively, while no difference was shown between the PSF and HCs.

**Conclusions:**

This study highlights the use of AL index in detecting early psychiatric risk. Distinct physiological patterns were observed in the PSF and CF groups. A targeted 10-biomarker AL index maintained predictive accuracy.

**Disclosure of Interest:**

None Declared

